# Molecular detection of *Ehrlichia spp.* in blood samples of dogs in southern Iran using polymerase chain reaction

**Published:** 2017-12-15

**Authors:** Noushin Derakhshandeh, Hassan Sharifiyazdi, Mohammad Abbaszadeh Hasiri

**Affiliations:** *Department of Clinical Sciences, Faculty of Veterinary Medicine, Shiraz University, Shiraz, Iran.*

**Keywords:** Dog, *Ehrlichia*, Epidemiology, Iran, Polymerase chain reaction

## Abstract

Ehrlichiosis is a zoonotic disease which has been reported from some regions of Iran. This study was aimed to determine the presence and prevalence of ehrlichiosis in suspected dogs referred to the Faculty of Veterinary Medicine, Shiraz University, Shiraz, Iran using polymerase chain reaction (PCR). Blood samples were collected from 98 suspected dogs with at least one of the five following findings: thrombocytopenia, anemia (hematocrit < 37.00%), gastrointestinal signs and respiratory and/or central nervous system diseases. Complete blood count was performed for each sample. After genomic DNA extraction, PCR assay was carried out using a commercial PCR kit. The results showed that only three out of 98 samples (3.06%) were positive for ehrlichiosis. There was no significant difference in hematological parameters between infected and non-infected cases. These results emphasize that ehrlichiosis has a low prevalence among examined cases in southern Iran. Further serological and molecular studies are needed to clarify the epidemiological feature of this infection in different areas of Iran.

## Introduction

Canine ehrlichiosis is a tick borne disease caused by gram-negative, obligate intracellular, pleomorphic and tick-borne bacteria.^1^ In 1935, canine monocytic ehrlichiosis was first reported by Dontien and Lestoquar.^2^ Then, in the United States canine granulocytic ehrlichiosis (caused by *Ehrlichia ewingii*) was branded in 1971.^3^

Transmission of ehrlichia can occur through blood sucking arthropods such as the tick *Rhipicephalus sanguineus*.^4^ Clinical signs of ehrlichiosis can be variable, depending on strain variation, host immune response, stage of disease and the presence of concurrent infections. Clinical findings are lethargy, inappetence, fever, weight loss, generalized lymphadenopathy, ocular and nasal discharges, mucosal and cutaneous petechial and ecchymotic hemorrhages and neurological signs.^5^^,^^6^ The diagnosis of ehrlichiosis relies on cultivation, serology, direct microscopical examination and molecular techniques.^7^ Smear diagnosis has low sensitivity, as intra-cytoplasmic morulae can be visualized only during the acute phase.^8^ Serology cannot discriminate between a current and previous exposure to the pathogen and may show false negative results in acute ehrlichiosis. Therefore, serology is an unreliable tool for ehrlichiosis diagnosis.^5^ Polymerase chain reaction (PCR) using whole-blood sample is the most sensitive method for diagnosis of acute ehrlichiosis.^9^^,^^10^

The aim of the present study was to evaluate the prevalence of ehrlichiosis in dog populations in Fars province in the south of Iran. In addition, complete blood count and epidemiological features (gender, breed, age and type of maintenance) were evaluated to determine if there was an association between these features and ehrlichiosis infection.

## Materials and Methods

**Sample collection. **From September 2014 to June 2015, a number of 98 dogs referred to the Veterinary Teaching Hospital of Shiraz University, were assessed. The study was performed in Shiraz, Fars province (southern Iran). 

Blood samples were collected from cases with at least one of the five following findings: thrombocytopenia (platelet count ≥140,000 per μL), anemia (hematocrit <37.00%), gastrointestinal signs and respiratory and/or central nervous system diseases.

Complete history including age, breed, gender, clinical signs especially respiratory signs (sneezing, coughing and/or nasal discharge), gastrointestinal signs (vomiting or diarrhea), type of housing (outdoor or indoor), previous tick infestation and presence of the other dogs were taken. Clinical examinations including temperature, pulse, and respiratory rates, assessment of depression or fever and thoracic auscultation were performed. Blood sample was collected via cephalic vein puncture from each dog and stored in EDTA containing tubes.

Hematological examination was carried out using automatic cell counter (Exigo, Stockholm, Sweden). Then, blood smears were prepared for microscopic investigation of morulae using Giemsa stain. Finally, samples were kept frozen at – 20 ˚C until DNA extraction.

**Polymerase chain reaction. **Genomic DNA was extracted from 100 µL of anticoagulated whole blood using a commercial DNA extraction kit (Qiagen®, Hilden, Germany) according to the manufacturer’s instruction. In this study, molecular investigation was performed using a commercially available PCR kit (iNtRON, Daejeon, South Korea) for detection of *Ehrlichia* spp. in blood samples as previously described by Akhtardanesh *et al*.^11^

PCR reaction (20 µL) was contained i-starTaqTM DNA polymerase (2.5 U), dNTPs (250 mM each), 10X reaction-buffer, chemical stabilizer (1 X), gel loading buffer (1 X), 8-methoxypsoralen (8-MOP) (25 µg mL^-1^), primers (10 pmol each), DNA template (2 µL; ~40 ng) and DNase and RNase free water (18 µL). A PCR product of 336 bp in size was amplified using thermocycler (MG 5331; Eppendorf, Hamburg, Germany) with thermal conditions as follows: initial denaturation at 94 ˚C for 5 min, followed by 40 amplification cycles (94 ˚C for 30 sec, 52 ˚C for 30 sec and 72 ˚C for 40 sec) and a final extension cycle (72 ˚C for 5 min). The presence of PCR products and their molecular weights were evaluated by electrophoresis of 7 μL of each product in 1.50 % agarose gel stained with Red Safe dye (iNtRON) under ultraviolet illumination. Positive control (*Ehrlichia* spp. DNA was available in the commercial kit) and negative controls (distilled water and DNA from healthy dog) were used in each round of PCR. 

**Statistical analysis. **The prevalence values based on gender, different breeds, ages and maintenance type of dogs were compared by Fischer exact test. Hematological parameters of positive and control group dogs were compared by Student's *t* test. The data were analyzed using SPSS software (version 16.0; SPSS Inc., Chicago, USA).

## Results

The mean ages of population were two years. The 47.30% of population was younger than two years and 52.70% were older. In physical examinations, 13 dogs (26.50%) were tick-infested. Forty dogs (40.80%) were anemic (hematocrit < 37.00%) and 18 cases (18.90%) were thrombocytopenic. Also, 15 (17.20%), 34 (35.40%) and 8 (16.00%) dogs had general signs (fever and lethargy), gastrointestinal signs (vomiting or diarrhea) and nervous signs (seizure and paralysis), respectively.

**Fig. 1:**
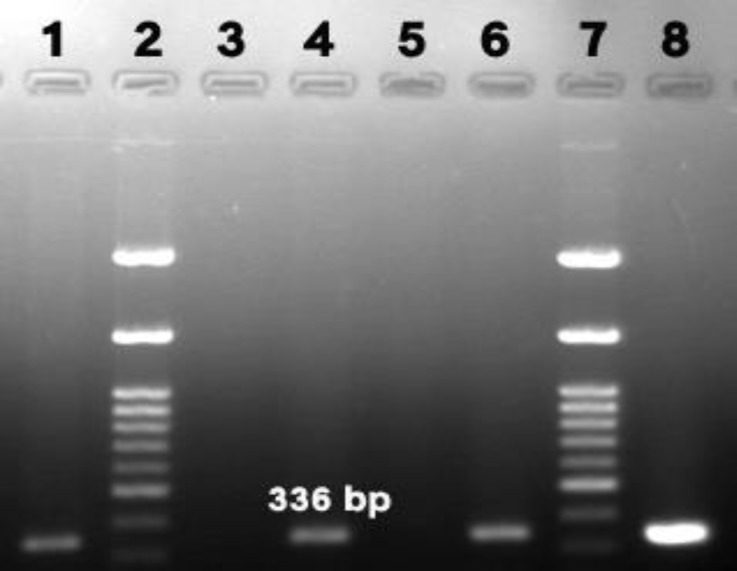
Agarose gel of PCR products (336 bp) obtained from positive cases for *Ehrlichia *spp. Lane 2 and 7 are molecular ladder lane 8 is positive control.

The results revealed that three samples (3.06%) out of 98 examined samples, were found positive using PCR (Fig. 1). However, in stained blood smears, no evidence of inclusion bodies or morulae formation was seen.

Higher prevalence of ehrlichiosis was recorded in females (7.90%) compared to males (0.00%). All *ehrlichia*-positive cases (n = 3) lived outdoor and were female. Two of positive dogs were mixed breeds and less than two years old. Two of positive cases housed adjacent to another dogs. Also, two cases of the positive dogs showed gastrointestinal sign (diarrhea) and one had neurological sign (seizure) on their history and physical examinations. In this study, two out of three *ehrlichia*-positive dogs had a history of tick infestation.

The seropositive dogs had a lower average count of red blood cells (RBC), (4.23 ×10^6^ per mL) and hematocrit (30.20%) compared to these values (RBC count: 5.18 ×10^6^ per mL and hematocrit: 36.45%) in negative cases, but these differences were not statistically significant.

## Discussion

For the first time, canine ehrlichiosis was reported in 1935 by Donatien and Lestoquard in Algeria in tick-infested dogs.^2^ During recent years, molecular identification of canine ehrlichiosis has been reported in dogs, both domestic and wild, in many parts of the world.^12^ In Iran, the first morphological evidence of possible dog infection with *Ehrlichia* was notified in Fars province.^13^ The present study is the first report of the molecular detection of ehrlichiosis in dog populations in southern Iran. The results revealed a low overall prevalence (3.06%) of *Ehrlichia* infection in this area.

Some species of canine *Erhrlichia* have also been proposed to be potential zoonoses.^14^^,^^15^ Therefore, the role of infected dogs as potential sources of infection for humans is important due to zoonotic aspects of disease, especially in immunocompromised persons.

The results of the present study showed that PCR is more sensitive than direct examination of stained blood smears for detecting ehrlichiosis in dogs. Morulae were not seen in none of the samples with clinical signs of disease and even in the PCR-positive cases, which is in agreement with other studies.^8^^,^^16^ *Ehrlichia* species multiply in the blood cells and form intracytoplasmic microcolonies called morulae during the acute phase of infection.^17^ Unfortunately, the observation of morulae is difficult and time-consuming and has been estimated to be successful in only about 4.00% of the cases.^18^ Previously, some studies also described that negative hematological results can occur in chronically infected dogs.^19^ Accordingly, PCR test has been suggested as a sensitive method for detection of organisms, especially in the chronic stage of infection.^20^^,^^21^

Some researchers reported that *E. canis* can infect all breeds of dogs.^22^ However, the German shepherd dogs showed more severe form of the disease with a higher morbidity and mortality compared to the other breeds. In this study, no breed predilection has been established for the German shepherd dog.

The reported prevalence of ehrlichiosis from other regions of Iran showed that Iran is an endemic country for this infection. In Tehran, blood samples from 240 dogs were analyzed by PCR and a prevalence of 16.50% was reported.^23^ In Kerman, blood samples were analyzed by indirect immunofluorescence antibody test (IFA) and rapid immunochromatography assay (ICA) to detect antibodies against *E. canis *in 123 apparently healthy dogs.^24^ The overall prevalence rate in their study was 14.63% and they found morulae formation only in three seropositive dogs (16.60%) during blood smear examinations. Ehrlichiosis have been reported in 9.60% of 198 companion seropositive dogs by means of IFA and ICA in Ahvaz. Morulae of *E. canis *were observed in monocyte of four infected dogs (2.02%).^25^ Also, a low seroprovalence of infection (0.80%) in North Khorasan of Iran (Mashhad) was determined using IFA test.^26^ In the present study, prevalence of ehrlichiosis was 3.060%. The mentioned researches have performed on both healthy and diseased cases, in contrast, in the present study only suspected cases were included in our molecular study for detection. So, the actual prevalence of detectable positive cases may be lower than 3.06%. The results showed that ehrlichiosis is not endemic in Fars province.

The variation in the prevalence of ehrlichiosis in different studies may be due to factors such as different diagnostic methods, sample size, geographical area, distribution of tick species and sampling season.^27^

Ticks act as mechanical vector of ehrlichiosis. The frequency of canine ehrlichiosis was higher in tick-infested dogs (two out of three cases) which is in agreement with studies of Costa *et al*. and Khazani *et al*.^28^^,^^29^ However, due to few positive cases in this study, infection rate was not statistically related to tick infestations (*p* > 0.05).

The brown dog tick, *Rhipicephalus sanguineus*, is the main vector for *Ehrlichia* in dogs. *Rhipicephalus sanguineus* is broadly distributed especially in tropical and sub-tropical areas of the world.^30^^,^^31^ Canine infestations by *R. sanguineus *in Fars province was also reported.^32^

In the present study, canine ehrlichiosis was observed only in female dogs which is in agreement with the research of Milanjeet *et al*.^27^ Nevertheless, it is not similar to Maazi *et al*. and Solano-Gallego *et al*. findings.^23^^,^^33^ The most positive cases of ehrlichiosis were observed in outdoor (3/3), younger (< 2years) (2/3), mix breeds (2/3) and female (3/3) dogs and in dogs that lived with other dogs (2/3). In the present study, the association of these risk factors and prevalence of ehrlichiosis was not significant. Studies of Akhtardanesh *et al*. and Maazi *et al*. showed that there were no relations between canine ehrlichiosis and age, breed, sex and type of dogs housing.^11^^,^^23^

The clinical sign of disease may vary based on infective strain, infectious dose, host’s breed, immunity of the infected dog and co-infection with other microorganisms. In the present study, neurological and gastrointestinal involvements were present in one positive case. It seems that neurological signs as a result of meningeal bleeding and/or meningitis can happen in the positive cases.^20^

The most common hematological abnormalities associated with ehrlichiosis are anemia and thrombo-cytopenia.^10^ In this study, anemia (hematocrit < 37.00%) and thrombocytopenia (platelet count ≥ 140,000 per μL) were observed in two of positive cases. Although hematological findings such as anemia, thrombocytopenia and leukopenia were more frequent in positive dogs, because of few positive cases, ehrlichiosis was not significantly related to hematological disorders. This result is in agreement with a number of previous studies.^34^^-^^36^ However, Milanjeet *et al*. showed that canine ehrlichiosis is related to significant hematological abnormalities.^27^

This is the first molecular detection of ehrlichiosis in dogs in southern Iran which highlights the need for continuous *Ehrlichia* spp. surveillance in Iran and assessing their effect on human health. Accordingly, further molecular investigations and sequencing are needed to clarify the epidemiological importance of disease and also further studies should be performed in other tick borne diseases like anaplasmosis and babesiosis in which co-infections with *Ehrlichia* may exist.
